# Associations between sarcopenia and circulating branched-chain amino acids: a cross-sectional study over 100,000 participants

**DOI:** 10.1186/s12877-024-05144-5

**Published:** 2024-06-21

**Authors:** HuiMin Liu, Qiang Zhang, QianMeng Hao, QingSheng Li, LingFei Yang, Xuan Yang, KaiXin Wang, JunFang Teng, Zhe Gong, YanJie Jia

**Affiliations:** 1 Department of Neurology, The First Affiliated Hospital of Zhengzhou University, Zhengzhou, 450052 Henan China; 2 School of Nursing and Health, Zhengzhou University, High-Tech Development Zone of States, 101 Kexue Road, Zhengzhou, NO China; 3 Department of Blood Transfusion, The Second Affiliated Hospital of Zhengzhou University, Zhengzhou, 450053 Henan China

**Keywords:** Branched-chain amino acid, Sarcopenia, Muscle mass, Hand grip strength, Mediation analysis

## Abstract

**Background:**

Emerging evidence suggests that alterations in BCAA metabolism may contribute to the pathogenesis of sarcopenia. However, the relationship between branched-chain amino acids (BCAAs) and sarcopenia is incompletely understood, and existing literature presents conflicting results. In this study, we conducted a community-based study involving > 100,000 United Kingdom adults to comprehensively explore the association between BCAAs and sarcopenia, and assess the potential role of muscle mass in mediating the relationship between BCAAs and muscle strength.

**Methods:**

Multivariable linear regression analysis examined the relationship between circulating BCAAs and muscle mass/strength. Logistic regression analysis assessed the impact of circulating BCAAs and quartiles of BCAAs on sarcopenia risk. Subgroup analyses explored the variations in associations across age, and gender. Mediation analysis investigated the potential mediating effect of muscle mass on the BCAA-muscle strength relationship.

**Results:**

Among 108,017 participants (mean age: 56.40 ± 8.09 years; 46.23% men), positive associations were observed between total BCAA, isoleucine, leucine, valine, and muscle mass (beta, 0.56–2.53; *p* < 0.05) and between total BCAA, leucine, valine, and muscle strength (beta, 0.91–3.44; *p* < 0.05). Logistic regression analysis revealed that increased circulating valine was associated with a 47% reduced sarcopenia risk (odds ratio = 0.53; 95% confidence interval = 0.3–0.94; *p* = 0.029). Subgroup analyses demonstrated strong associations between circulating BCAAs and muscle mass/strength in men and individuals aged ≥ 60 years. Mediation analysis suggested that muscle mass completely mediated the relationship between total BCAA, and valine levels and muscle strength, partially mediated the relationship between leucine levels and muscle strength, obscuring the true effect of isoleucine on muscle strength.

**Conclusion:**

This study suggested the potential benefits of BCAAs in preserving muscle mass/strength and highlighted muscle mass might be mediator of BCAA-muscle strength association. Our findings contribute new evidence for the clinical prevention and treatment of sarcopenia and related conditions involving muscle mass/strength loss.

**Supplementary Information:**

The online version contains supplementary material available at 10.1186/s12877-024-05144-5.

## Introduction

Sarcopenia is characterized by the progressive loss of muscle mass, strength, and function. It is a significant public health concern, particularly among older adults and is associated with an increased risk of falls, fractures, physical disability, and mortality [1, 2]. With the continuous increase in global life expectancy and the escalating trend of population aging, the prevalence of sarcopenia is expected to increase in the coming decades, posing a significant burden on healthcare systems worldwide [3]. Currently, there is a lack of clinically effective therapeutic drugs for sarcopenia, which presents a significant barrier to its treatment [4]. Therefore, it is imperative to explore effective preventive and treatment strategies for this disease.

Recently, there has been a growing interest in the potential role of branched-chain amino acids (BCAAs) in the development and progression of sarcopenia. BCAAs, including isoleucine, leucine, and valine, are essential amino acids that cannot be synthesized by the human body and must be obtained from dietary sources [5]. These amino acids play a vital role in muscle protein metabolism by acting as substrates for protein synthesis and regulators of muscle protein turnover [6–9].

Emerging evidence suggests that alterations in BCAA metabolism contribute to the pathogenesis of sarcopenia. Studies have reported lower levels of circulating BCAAs in individuals with sarcopenia compared to healthy individuals [10, 11], suggesting a potential protective role of BCAAs against sarcopenia [12–14]. Furthermore, some studies based on metabolomics techniques have found that circulating BCAAs are associated with muscle mass in functionally limited elderly individuals [15], poor muscle quality in the elderly [16], muscle function in older Japanese women [17], and sarcopenia in community-dwelling older adults [18]. Despite these findings, the relationship between BCAAs and sarcopenia-related traits is incompletely understood, and existing literature presents conflicting results. A meta-analysis reported a positive association between BCAA levels and muscle strength [11], consistent with a previous study that investigated the effects of complex protein supplements on muscle strength [19]. However, this contradicts the findings of other meta-analyses that reported no significant differences between BCAA levels and handgrip strength in individuals aged > 60 years [20, 21] and non-frail older individuals [22]. Although these studies did not control for important confounders (e.g., socioeconomic status and education score); were limited by small sample sizes; did not discuss the differences by age and gender; mainly focused on the population aged > 60 years; and had inconsistent results, they suggested a potential link between BCAAs and sarcopenia-related traits (muscle mass/strength).

In this present study, we conducted the largest-scale community-based cohort study, enrolling over 100,000 adults from the United Kingdom (UK), to comprehensively investigate the association between circulating BCAAs (i.e., total BCAA, isoleucine, leucine, and valine) and muscle mass/strength. Previous study demonstrated that wasting protein energy can cause a decline in muscle mass, leading to reduced strength [23]. Thus, we employed mediation analysis [24] for the first time, considering the potential mediating role of muscle mass in the association between circulating branched-chain amino acids and muscle strength. This study presents novel evidence contributing to the clinical understanding and management of sarcopenia and other conditions linked to the depletion of muscle mass and strength.

## Materials and methods

### Participants

**Fig. 1:**
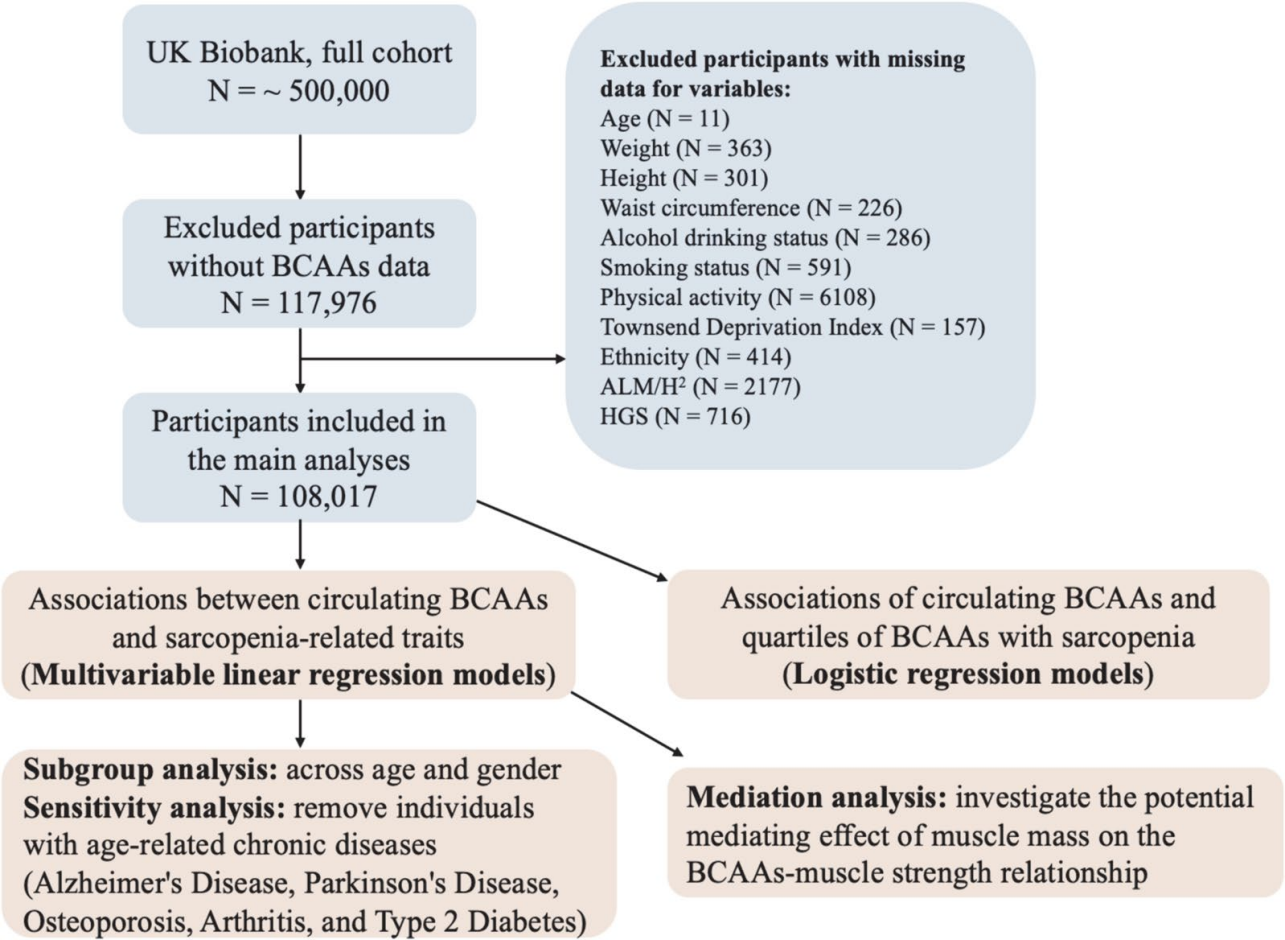
Flowchart of the study. The number of participants (N) in the population enrollment process. BCAAs, Branched-chain amino acids; ALM/H^2^, appendicular lean mass divided by height squared; HGS, hand grip strength

This cross-sectional analysis utilized data from the UK Biobank cohort, an ongoing multicenter study comprising approximately 500,000 individuals aged 38–73 years between 2006 and 2010. Participants were recruited from 22 centers across England, Scotland, and Wales for initial assessments. Clinical and biochemical information was obtained through touchscreen questionnaires, physical examinations, sample collection, and electronic health records within the UK Biobank cohort. Ethical approval was obtained from the North-west Multicenter Research Ethics Committee, and all participants provided written informed consent. Additional details about the UK Biobank project can be found in other published work [25]. Among the 117,976 participants with available BCAAs data, 9,959 participants with missing data for at least one variable were excluded, resulting in a final analysis cohort of 108,017 participants. A flowchart of the study is presented in Fig. 1.

### BCAA quantification

BCAA quantification followed established protocols described in previous publications [26–29]. EDTA plasma samples were collected from the UK Biobank (UKB), consisting of 118,000 samples at baseline and additional 5,000 samples for repeat assessment. These samples were transported to the Nightingale Health Laboratories in Finland and analyzed between June 2019 and April 2020 using a high throughput targeted nuclear magnetic resonance metabolomics platform (Nightingale Health Plc; biomarker quantification version 2020). This platform enabled the simultaneous quantification of 249 metabolic measures, including the absolute concentrations of various metabolites and their ratios. Metabolites were measured following standard biochemical analysis protocols with laboratory staff blinded to the associated health outcomes. Subsequently, in late May 2020, the metabolomic data were compiled and linked to clinical information in the UK Biobank. The dataset was submitted to the UK Biobank Research Ethics Committee in March 2021. In this study, the BCAAs included total BCAA, isoleucine, leucine, and valine. The circulating concentrations of total BCAA (UKB Field 23,464), isoleucine (UKB Field 23,465), leucine (UKB Field 23,466), and valine (UKB Field 23,467) were extracted from the UK Biobank dataset. The circulating concentration of total BCAA was equal to the sum of the circulating concentrations of isoleucine, leucine, and valine.

### Measurement of sarcopenia-related traits

This study used hand grip strength (HGS) (UKB Fields 46 and 47) and appendicular lean mass (ALM) as physical capability markers to define sarcopenia [1]. HGS was measured using a Jamar J00105 hydraulic hand dynamometer, with one measurement recorded for each hand. The average HGS, expressed in kilograms (kg), was used for subsequent analysis. ALM was estimated using the Janssen equation [30] applied to the total body composition measured by bioimpedance analysis (BIA) using a Tanita BC418MA device from Tokyo, Japan. The validity of BIA-measured ALM was evaluated by comparing it to muscle mass measured using dual-energy X-ray absorptiometry in a subset of UK Biobank participants, as previously reported, using Pearson’s correlation coefficient and a Bland–Altman plot [31]. ALM was calculated as the sum of muscle mass in the upper and lower limbs (UKB Field 23,113, 23,117, 23,121, and 23,125). To account for variations in body size, ALM was divided by height squared (ALM/H^2^, kg/m^2^) [1, 2]. Following the criteria set by the European Working Group on Sarcopenia in Older People (EWGSOP2) [1], HGS was categorized into normal (≥ 27 kg for men; ≥ 16 kg for women) and low (< 27 kg for men; < 16 kg for women) groups, while ALM/H^2^ was categorized into normal (≥ 7 kg/m^2^ for men; ≥ 5 kg/m^2^ for women) and low (< 7 kg/m^2^ for men; < 5 kg/m^2^ for women) groups. According to the diagnostic criteria recommended by the EWGSOP2 [1], participants with low muscle strength in present study were defined as sarcopenia.

### Covariates

Demographic information, including age (UKB Field 21,003), gender (UKB Field 31), height (UKB Field 50), and waist circumference (UKB Field 48), was collected during the initial assessment. To account for the small sample size of the underweight group, it was combined with the normal-weight group for the analysis. Smoking (UKB Field 20,116) and alcohol consumption status (UKB Field 20,117) were self-reported as “never,” “previously,” or “currently.” Physical activity (UKB Field 884) was assessed as the number of days per week of moderate physical activity lasting at least 10 min. Education scores (UKB Fields 26,414, 26,421, and 26,431) were self-reported. Ethnicity (UKB Field 21,000) was captured to reflect longitudinal variation in living experiences and categorized as White (including British, White, Irish, or any other White background), Black (including Black or Black British, White and Black Caribbean, White and Black African, African, Caribbean, or any other Black background), Asian (including Asian or Asian British, Chinese, Indian, Pakistani, Bangladeshi, or any other Asian background), or multiracial (including mixed, any other mixed background, or other ethnic groups). The Townsend Deprivation Index (UKB Field 189), calculated using the national census data, categorized participants into high (values below -2.08), middle (values ranging from -2.08 to 1.40), or low deprivation (values above 1.40) levels based on factors such as unemployment, vehicle ownership, household overcrowding, and occupation.

### Statistical analysis

The participants were categorized into two groups based on the presence or absence of sarcopenia. In this study, we conducted Kolmogorov–Smirnov tests on all continuous variables, and the results indicated that they all met the assumption of normality (eTable 1, *p* values > 0.05). Therefore, statistical tests, including t-tests for continuous variables and chi-squared tests for categorical variables, were employed for comparisons.

Multiple linear regression analysis [32, 33] was conducted to assess the relationship between circulating BCAAs (total BCAA, isoleucine, leucine, and valine) and sarcopenia-related traits (ALM/H^2^ was used for muscle mass and HGS for muscle strength). The estimated changes in ALM/H^2^ and HGS per unit increase in circulating BCAAs are represented by beta coefficients. Logistic regression analysis [34, 35] was performed to evaluate the impact of a one-unit increase in circulating BCAAs and quartiles of circulating BCAAs (using Q1 as the reference level) on sarcopenia risk, expressed as odds ratios (ORs). Appropriate measures were taken to account for influential confounding factors. Models 1 and 2 were utilized, with model 1 adjusted for age, gender, and BMI, and model 2 adjusted for confounding factors such as smoking, alcohol consumption, physical activity, ethnicity, education score, and Townsend Deprivation Index. The primary findings were derived from model 2. Prior to the regression analyses, a multicollinearity assessment was conducted for the independent variables in model 2, with a variance inflation factor (VIF) of < 2.5, indicating the absence of multicollinearity. Subgroup analyses were performed to examine potential variations in the associations between circulating BCAAs and ALM/H^2^, as well as HGS, across different subgroups defined by age (< 60 and ≥ 60 years, based on established criteria for defining an older person by the United Nations) and gender (male and female). In the sensitivity analysis, we excluded individuals with age-related chronic diseases (Alzheimer's Disease, Parkinson's Disease, Osteoporosis, Arthritis, and Type 2 Diabetes) and reanalyzed the association between BCAAs concentrations (total BCAA, isoleucine, leucine, and valine) and muscle mass/strength. Finally, we conducted a mediation analysis [24] to investigate the potential mediating effect of muscle mass (ALM/H^2^) on the relationship between circulating BCAAs and muscle strength (HGS). Subgroup analysis, sensitivity analysis, and mediation analysis all adjusted for confounding factors in Model 2. Statistical significance was set at *p* < 0.05.

The R software version 4.0.2 was utilized for analysis. The “stats” package was employed for multivariable linear regression and logistic regression analyses; the “mediation” package was used for causal mediation analysis; and the “forest plot” package was utilized for generating the forest plot.

## Results

### Baseline characteristics

Of the recruited 108,207 participants, 49,941 (46.23%) were men. The mean (standard deviation [SD]) age of participants was 56.40 (8.09) years. Compared with participants with non-sarcopenia, those with sarcopenia were more likely to be older, female, non-drinkers, less physically activity, highly educated, lower Townsend Deprivation Index and Asian. In our study, without adjusting for confounding factors, we observed higher levels of total BCAA, and leucine in the sarcopenia group than in the non-sarcopenia group (*p* values < 0.05). However, the two groups had no significant difference in the isoleucine and valine levels (p values > 0.05). Participants’ characteristics are presented in Table 1.

**Table 1:** Characteristics of participants

Characteristics	All participates (*N* = 108,017)	Sarcopenia (*N* = 8895)	Non-sarcopenia (*N* = 99,122)	p
Age, years (mean, SD)	56.40 (8.09)	59.81 (7.18)	56.10 (8.10)	< 0.001
Age category, N (%)				< 0.001
< 60 years	61,961 (57.36)	3505 (39.40)	58,456 (58.97)	
≥ 60 years	46,056 (42.64)	5390 (60.60)	40,666 (41.03)	
Gender, N (%)				< 0.001
Male	49,941 (46.23)	3208 (36.07)	46,733 (47.15)	
Female	58,076 (53.77)	5687 (63.93)	52,389 (52.85)	
Waist circumference, cm (mean, SD)	90.13 (13.33)	91.20 (13.89)	90.04 (13.28)	< 0.001
Alcohol drinking status, N (%)				< 0.001
Never	4327 (4.01)	741 (8.33)	3586 (3.62)	
Previous	3709 (3.43)	523 (5.88)	3186 (3.21)	
Current	99,981 (92.56)	7631 (85.79)	92,350 (93.17)	
Smoking status, N (%)				0.057
Never	59,125 (54.74)	4965 (55.82)	54,160 (54.64)	
Previous	37,737 (34.94)	3006 (33.79)	34,731 (35.04)	
Current	11,155 (10.33)	924 (10.39)	10,231 (10.32)	
Physical activity, day (mean, SD)	3.63 (2.33)	3.44 (2.45)	3.65 (2.32)	< 0.001
Townsend Deprivation Index, N (%)				< 0.001
Low	20,380 (18.87)	2264 (25.45)	18,116 (18.28)	
Middle	9629 (8.91)	900 (10.12)	8729 (8.81)	
High	78,008 (72.22)	5731 (64.43)	72,277 (72.92)	
Education score (mean, SD)	13.66 (15.78)	16.82 (17.96)	13.37 (15.53)	< 0.001
Ethnicity, N (%)				< 0.001
White	102,812 (95.18)	8131 (91.41)	94,681 (95.52)	
Black	1527 (1.41)	117 (1.32)	1410 (1.42)	
Asian	2203 (2.04)	479 (5.38)	1724 (1.74)	
Multiracial	1475 (1.37)	168 (1.89)	1307 (1.32)	
ALM/H^2^, kg/m^2^ (mean, SD)	8.25 (1.36)	8.08 (1.37)	8.27 (1.36)	< 0.001
HGS, kg (mean, SD)	31.02 (11.03)	15.83 (5.86)	32.38 (10.34)	< 0.001
Isoleucine, mmol/L (mean, SD)	0.05 (0.02)	0.050 (0.018)	0.050 (0.017)	0.213
Isoleucine category, N (%)				0.207
Q1	27,007 (25)	2160 (24.28)	24,847 (25.07)	
Q2	27,002 (25)	2283 (25.67)	24,719 (24.94)	
Q3	27,004 (25)	2197 (24.70)	24,807 (25.03)	
Q4	27,004 (25)	2255 (25.35)	24,749 (24.97)	
Leucine, mmol/L (mean, SD)	0.10 (0.03)	0.100 (0.028)	0.101 (0.028)	< 0.001
Leucine category, N (%)				< 0.001
Q1	27,005 (25)	2416 (27.16)	24,589 (24.81)	
Q2	27,007 (25)	2242 (25.21)	24,765 (24.98)	
Q3	27,001 (25)	2096 (23.56)	24,905 (25.13)	
Q4	27,004 (25)	2141 (24.07)	24,863 (25.08)	
Valine, mmol/L (mean, SD)	0.20 (0.04)	0.202 (0.043)	0.203 (0.042)	0.203
Valine category, N (%)				0.390
Q1	27,009 (25)	2283 (25.66)	24,726 (24.95)	
Q2	27,011 (25)	2227 (25.04)	24,784 (25.00)	
Q3	27,001 (25)	2173 (24.43)	24,828 (25.05)	
Q4	26,996 (25)	2212 (24.87)	24,784 (25.00)	
Total BCAA, mmol/L (mean, SD)	0.35 (0.08)	0.352 (0.086)	0.354 (0.084)	0.021
Total BCAA category, N (%)				0.011
Q1	27,007 (25)	2314 (26.01)	24,693 (24.91)	
Q2	27,008 (25)	2251 (25.31)	24,757 (24.98)	
Q3	26,998 (25)	2105 (23.67)	24,893 (25.11)	
Q4	27,004 (25)	2225 (25.01)	24,779 (25.00)	

The concentration of total branched-chain amino acid (BCAA) is equal to the sum of the concentrations of isoleucine, leucine and valine

*Abbreviations*: *SD* standard deviation, *N* number, *ALM/H*^*2*^ appendicular lean mass divided by height squared, *HGS* hand grip strength

### Associations between BCAAs and sarcopenia-related traits

**Fig. 2:**
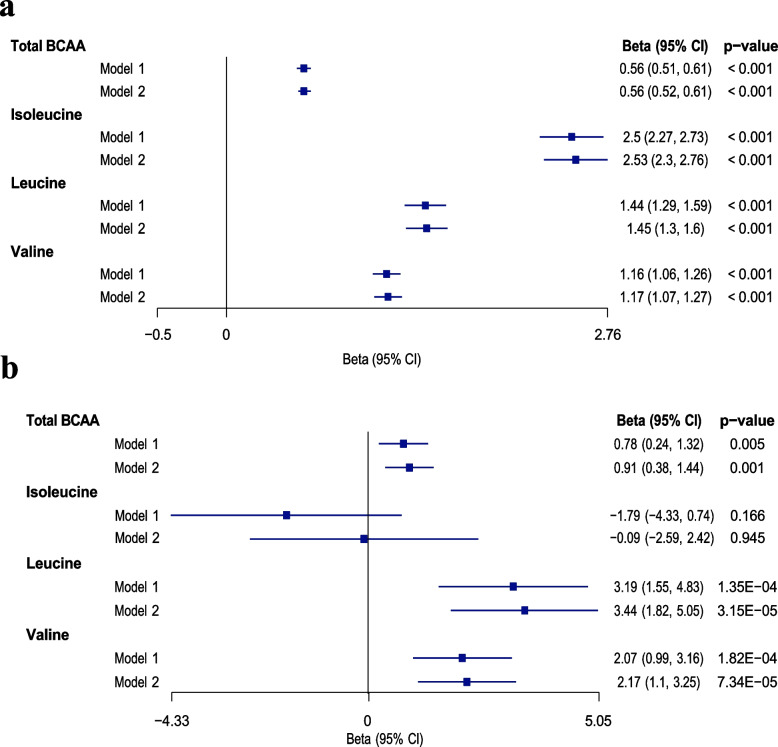
Associations between circulating BCAAs and ALM/H^2^ (**a**) and HGS (**b**). The estimated changes in ALM/H^2^ and HGS per unit increase in circulating BCAAs are represented by beta coefficients (95% CI). Model 1 was adjusted for age, gender, and waist circumference, and model 2 was additionally adjusted for confounding factors such as smoking status, alcohol consumption status, physical activity, ethnicity, education score, and Townsend Deprivation Index. The primary findings were derived from model 2. CI, confidence interval; total BCAA, total branched-chain amino acid; ALM/H^2^, appendicular lean mass divided by height squared; HGS, hand grip strength

Our study demonstrated a positive association between all BCAAs and ALM/H^2^ in models 1 and 2 (Fig. 2). No significant multicollinearity was found among the covariates (VIF < 2.5, eTables 2–5). Every unit increase in circulating BCAAs corresponded to a significant increase in ALM/H^2^ by 0.56–2.53 kg/m^2^ (*p* < 0.05, Fig. 2a). The correlation between circulating BCAAs and ALM/H^2^ decreased in the following order: isoleucine, leucine, valine, and total BCAA (*p* < 0.05, Fig. 2a). Furthermore, inconsistent associations were observed between circulating BCAAs and HGS (*p* < 0.05, Fig. 2b). Total BCAA, leucine and valine were positively correlated, while isoleucine showed no insignificant correlation with HGS (Fig. 2b).

**Fig. 3:**
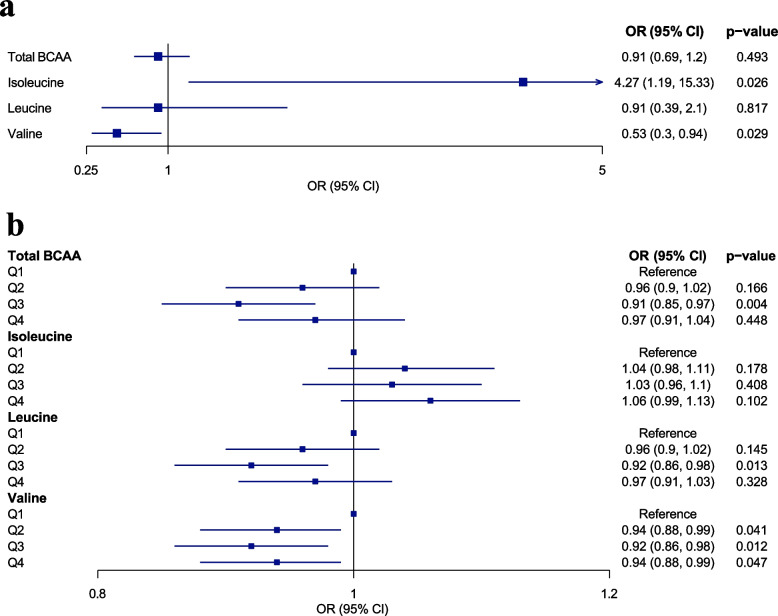
Associations of continuous BCAAs and quartiles of BCAAs with sarcopenia. Effect of a one-unit increase in circulating BCAAs (**a**) and the quartiles of circulating BCAAs (using Q1 as the reference level) (**b**) on sarcopenia risk, expressed as odds ratios (ORs). The model was adjusted for age, gender, waist circumference, smoking status, alcohol consumption status, physical activity, ethnicity, education score, and Townsend Deprivation Index. CI, confidence interval; BCAA, branched-chain amino acid

Moreover, we employed logistic regression to examine the impact of circulating BCAAs and BCAA concentration quartiles on the risk of sarcopenia (Fig. 3). Results revealed that only circulating valine consistently exhibits protective effects against sarcopenia. Each unit increase in circulating valine concentration was linked to a 47% decrease in the risk of sarcopenia (OR = 0.53, 95% confidence interval [CI] = 0.3–0.94, *p* = 0.029). Our study did not reveal a dose–response relationship between circulating valine levels and sarcopenia risk. As the valine concentration increased from Q2 to Q4, the risk of sarcopenia decreased by 6%, 8%, and 6%, respectively.

### Subgroup analyses

In the age-specific analyses, we observed a positive association between circulating BCAAs and ALM/H^2^ in age groups < 60 and ≥ 60 years (p < 0.05, eFigure 1a). However, there was a notable disparity in the associations between circulating BCAAs and HGS in age groups < 60 and ≥ 60 years (eFigure 1b). In the group aged ≥ 60 years, we found a positive correlation between circulating total BCAA, leucine, and valine and HGS, while no correlation was observed with isoleucine (eFigure 1b). In the group aged < 60 years, only leucine was found to be associated with HGS (eFigure 1b).

In the gender-specific analysis, our results indicated that the relationship between circulating BCAAs and ALM/H^2^, as well as HGS, in men, is consistent with that observed in the overall population (eFigure 2). However, we observed notable differences in the association between circulating BCAAs and sarcopenia-related traits, particularly HGS, in women compared to the overall population (eFigure 2b). Our study revealed a significant negative association between isoleucine levels and HGS in women (eFigure 2b). Additionally, we did not observe any association between circulating concentrations of total BCAA, leucine, valine and HGS in women (eFigure 2b).

### Sensitivity analysis

The sensitivity analysis results demonstrate that after excluding individuals with age-related chronic diseases (Alzheimer's Disease, Parkinson's Disease, Osteoporosis, Arthritis, and Type 2 Diabetes), the relationship between BCAAs concentrations and muscle mass/strength remains consistent with the results before exclusion (eFigure 3).

### Mediation analysis

**Fig. 4:**
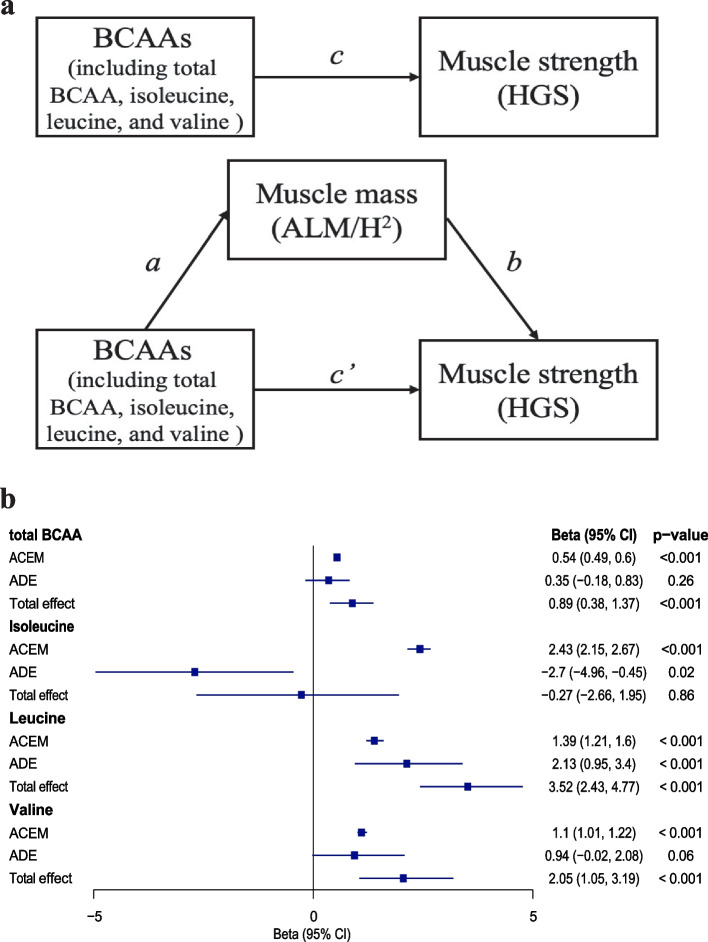
Theoretical framework (**a**) and results (**b**) of the mediation analysis. In Fig. 4a, the symbol “c” represents the total effect of BCAAs on muscle strength (HGS), “c” represents the average direct effect (ADE) of BCAAs on muscle strength, and “a × b” represents the average causal mediation effect (ACME) of BCAAs on muscle strength through muscle mass (ALM/H^2^). In Fig. 4b, beta was adjusted for age, gender, waist circumference, smoking status, alcohol consumption status, physical activity, ethnicity, education score, and Townsend Deprivation Index. BCAAs, branched-chain amino acids; ALM/H^2^, appendicular lean mass divided by height squared; HGS, hand grip strength; CI, confidence interval

Figure 4 presents the theoretical framework and results of the mediation analysis. In Fig. 4a, the symbol “c” represents the total effect of BCAAs on muscle strength (HGS), “c'” represents the average direct effect (ADE) of BCAAs on muscle strength, and “a × b” represents the average causal mediation effect (ACME) of BCAAs on muscle strength through muscle mass (ALM/H^2^). In this study, mediation analysis revealed that muscle mass acted as a complete mediator in the relationship between total BCAA, valine and muscle strength (p values of total effect < 0.05, p values of ACME < 0.05, and p values of ADE > 0.05) (Fig. 4b). Additionally, our results suggested that muscle mass might acted as a partial mediator in the relationship between leucine and muscle strength (p values of total effect < 0.05, p values of ACME < 0.05, and p values of ADE < 0.05) (Fig. 4b). Although isoleucine did not exhibit a significant total effect on muscle strength (beta = -0.27, 95% CI = -2.66 to 1.95), it had a positive effect on muscle strength when mediated by muscle mass (ACME: beta = 2.43, 95% CI = 2.15–2.67, p < 0.001), while also having a direct negative effect on muscle strength (ACME: beta = -2.7, 95% CI = -4.96 to -0.45, *p* = 0.02) (Fig. 4b). Our findings indicated that muscle mass may obscure the true association between isoleucine intake and muscle strength.

## Discussion

Using the largest-scale community-based cohort study, enrolling over 100,000 adults from the UK Biobank, we discovered significant associations between elevated levels of circulating BCAAs, including total BCAA, isoleucine, leucine, and valine, and increased muscle mass and strength. Furthermore, our findings revealed that higher circulating valine levels were linked to a reduced risk of sarcopenia; however, this relationship did not demonstrate a dose–response pattern. Subgroup analysis indicated that the association between circulating BCAA and muscle mass and strength was more prominent among men and individuals aged ≥ 60 years. Finally, our mediation analysis revealed novel findings, indicating that muscle mass potentially serves as a complete mediator in the pathway connecting total BCAA and valine to muscle strength. Additionally, it acts as a partial mediator in the pathway linking leucine to muscle strength and obscures the true effect of isoleucine on muscle strength.

The evidence from metabolomics study indicates a positive correlation between BCAAs and muscle mass in functionally limited older adults [15]. Furthermore, results from systematic reviews and meta-analyses suggest that BCAAs supplementation is beneficial for improving muscle mass in older adults [11]. This present study extends these findings to the middle-aged population (average age: 56 years), as we observed significant positive associations between total BCAAs, leucine, isoleucine, and valine with muscle mass. To elucidate these relationships, it has been postulated that overnight fasting leads to elevated serum BCAAs concentrations, which are proportional to the body's muscle mass [36]. BCAAs increase protein synthesis and decrease protein degradation in human muscles [7, 37]. BCAA supplementation can prevent the breakdown of proteins in skeletal muscles [8, 38]. However, the association between BCAA supplementation and grip strength remains controversial. Recent meta-analyses have yielded conflicting conclusions, with one suggesting that BCAA supplementation effectively enhanced grip strength in older adults and another indicating no improvement in grip strength among middle-aged to older adults. Our findings revealed positive associations between circulating BCAAs and grip strength; however, muscle mass likely influences or obscures this relationship. An adequate nutritional status is associated with maintaining muscle mass, which helps maintain muscle strength. These associations are cause-and-effect relationships that have been demonstrated in studies involving different populations [23, 39]. The underlying mechanism by which muscle mass mediates the association between BCAAs and muscle strength deserves more basic and clinical research.

In addition, the present study found that the association between circulating BCAAs and muscle mass and strength was significantly affected by gender and age. In the gender-stratified analysis, men showed a stronger association between circulating BCAAs and muscle mass (e.g., total BCAA, isoleucine, leucine, and valine) and strength (e.g., total BCAA, leucine, and valine) than women. Men were more likely to have greater skeletal muscle mass than women, and women tended to have a greater percentage of body fat than men, as confirmed by previous studies [40, 41]. The observed disparity in muscle mass between men and women can be attributed to several factors. First, higher levels of circulating androgens, particularly testosterone, are pivotal in promoting muscle protein synthesis and enhancing muscle growth [42, 43]. Second, men generally possess a greater proportion of type II muscle fibers [44], which is associated with increased muscle strength and hypertrophy. In the age-stratified analysis, we observed no significant difference in the association between circulating BCAA concentrations and muscle mass between the groups aged ≥ 60 and < 60 years. However, a notable disparity was evident in the association between circulating BCAAs and muscle strength between the two groups. Higher levels of circulating total BCAA, leucine, and valine were positively correlated with muscle strength in the group aged ≥ 60 years, while no association was found between any specific BCAA and muscle strength in the group aged < 60 years. Our results are consistent with those of a recent meta-analysis [11]. We speculated that the main reasons for this are as follows: First, aging is associated with a decline in anabolic hormone levels, such as testosterone [45], and growth hormone [46], which play crucial roles in muscle protein synthesis and maintenance. Consequently, older adults may excessively rely on alternative pathways for muscle protein synthesis, with BCAAs as important substrates. Second, older adults may experience greater muscle protein breakdown owing to factors such as chronic inflammation and oxidative stress [47], making the role of BCAAs in muscle maintenance and repair even more significant. In addition, the decline in physical activity levels and reduced muscle mass commonly observed with aging may lead to a higher reliance on BCAAs for muscle health. Collectively, these factors contribute to a stronger association between BCAAs and muscle mass and strength in older men than in younger women. However, further research is needed to fully elucidate the underlying mechanisms.

This study had several strengths. First, we used a large sample size to examine the association between circulating BCAA levels and muscle mass and strength, which enhanced the reliability of the study results. Second, by assessing circulating BCAA levels rather than relying on dietary BCAA concentrations, this study partly mitigated the influence of inter-individual variations in BCAA absorption and utilization, leading to more accurate and objective findings. Third, for the first time, our utilization of mediation analysis has underscored the crucial role of muscle mass in the association between BCAAs and muscle strength, thereby facilitating the discovery of a true relationship between BCAAs and muscle strength. However, this study had certain limitation. Although BCAAs are a group of essential amino acids that can only be obtained exogenously, the results of this study suggest the potential benefits of BCAA supplementation in improving muscle mass and strength. The study’s cross-sectional design precluded the establishment of a causal relationship between circulating BCAAs and muscle indices. Additionally, the smaller sample size in the sarcopenia group may impact the generalizability of our finding. Therefore, we performed rigorous statistical analyses, including subgroup analyses and sensitivity analyses, to assess the robustness of our results. However, it is essential for future research to include a more balanced representation of participants across these categories to enhance the validity and generalizability of the conclusions drawn from such investigations.

This study, based on a cohort of nearly 100,000 individuals, revealed that higher levels of circulating BCAAs were associated with greater muscle mass and strength, particularly in men and adults aged ≥ 60 years. Furthermore, muscle mass may mediate or confound the true association between circulating BCAAs and muscle strength. Further prospective cohort studies, basic research, and clinical trials are needed to confirm the association between circulating BCAAs and muscle mass and strength. Additionally, these investigations should provide evidence for developing interventions and treatments for sarcopenia and other conditions contributing to a decline in muscle function.

### Supplementary Information

Supplementary Material 1.

## Data Availability

This study was conducted using the UK Biobank software. The UK Biobank is an open-access resource, and bonafide researchers can access the UK Biobank dataset by registering at https://biobank.ndph.ox.ac.uk/showcase/. Additional information is available from the corresponding author upon request.
