# Supplementary material for: Associations between sarcopenia and circulating branched-chain amino acids: a cross-sectional study over 100,000 participants
Source: BMC Geriatr. 2024 Jun 21;24:541. doi: 10.1186/s12877-024-05144-5 (PMC11193178; doi:10.1186/s12877-024-05144-5)
Supplement: Supplementary file 1 — Supplementary Material 1. [file 12877_2024_5144_MOESM1_ESM.docx]

**eTable 1 Kolmogorov-Smirnov tests on all continuous variables**

| Variables | D value | p value |
| --- | --- | --- |
| Age | 0.539 | 0.933 |
| Waist circumference | 0.523 | 0.948 |
| Physical activity | 0.506 | 0.960 |
| Education score | 0.649 | 0.794 |
| ALM/H^2^ | 0.539 | 0.933 |
| HGS | 0.562 | 0.910 |
| Isoleucine | 0.565 | 0.907 |
| Leucine | 0.583 | 0.886 |
| Valine | 0.566 | 0.906 |
| Total BCAA | 0.551 | 0.922 |

Note: The concentration of total branched-chain amino acid (BCAA) is equal to the sum of the concentrations of isoleucine, leucine and valine. Abbreviations: ALM/H^2^, appendicular lean mass divided by height squared; HGS, hand grip strength;

**eTable 2 Multicollinearity assessment for independent covariates and total BCAA in model 2**

| Variables | VIF (model 2) |
| --- | --- |
| total BCAA | 1.13 |
| Sex | 1.31 |
| Waist circumference | 1.41 |
| Age | 1.04 |
| Alcohol drinking status | 1.06 |
| Smoking status | 1.04 |
| Physical activity | 1.02 |
| Townsend Deprivation Index | 1.27 |
| Ethnicity | 1.07 |
| Education score | 1.25 |

Model 2 adjusted age, gender, waist circumference, smoking status, alcohol drinking status, physical activity, ethnicity, education score, and Townsend Deprivation Index. total BCAA, total branched-chain amino acid; VIF, variance inflation factor. VIF less than 2.5 indicating the absence of multicollinearity.

**eTable 3 Multicollinearity assessment for independent covariates and isoleucine in model 2**

| Variables | VIF (model 2) |
| --- | --- |
| Isoleucine | 1.08 |
| Sex | 1.3 |
| BMI | 1.38 |
| Age | 1.04 |
| Alcohol drinking status | 1.06 |
| Smoking status | 1.04 |
| Physical activity | 1.03 |
| Townsend Deprivation Index | 1.27 |
| Ethnicity | 1.07 |
| Education score | 1.25 |

Model 2 adjusted age, gender, waist circumference, smoking status, alcohol drinking status, physical activity, ethnicity, education score, and Townsend Deprivation Index. VIF, variance inflation factor. VIF less than 2.5 indicating the absence of multicollinearity.

**eTable 4 Multicollinearity assessment for independent covariates and leucine in model 2**

| Variables | VIF (model 2) |
| --- | --- |
| Leucine | 1.13 |
| Sex | 1.33 |
| BMI | 1.39 |
| Age | 1.04 |
| Alcohol drinking status | 1.06 |
| Smoking status | 1.04 |
| Physical activity | 1.02 |
| Townsend Deprivation Index | 1.27 |
| Ethnicity | 1.07 |
| Education score | 1.25 |

Model 2 adjusted age, gender, waist circumference, smoking status, alcohol drinking status, physical activity, ethnicity, education score, and Townsend Deprivation Index. VIF, variance inflation factor. VIF less than 2.5 indicating the absence of multicollinearity.

**eTable 5 Multicollinearity assessment for independent covariates and valine in model 2**

| Variables | VIF (model 2) |
| --- | --- |
| Valine | 1.13 |
| Sex | 1.3 |
| BMI | 1.43 |
| Age | 1.04 |
| Alcohol drinking status | 1.06 |
| Smoking status | 1.04 |
| Physical activity | 1.03 |
| Townsend Deprivation Index | 1.27 |
| Ethnicity | 1.07 |
| Education score | 1.25 |

Model 2 adjusted age, gender, waist circumference, smoking status, alcohol drinking status, physical activity, ethnicity, education score, and Townsend Deprivation Index. VIF, variance inflation factor. VIF less than 2.5 indicating the absence of multicollinearity.

| a | 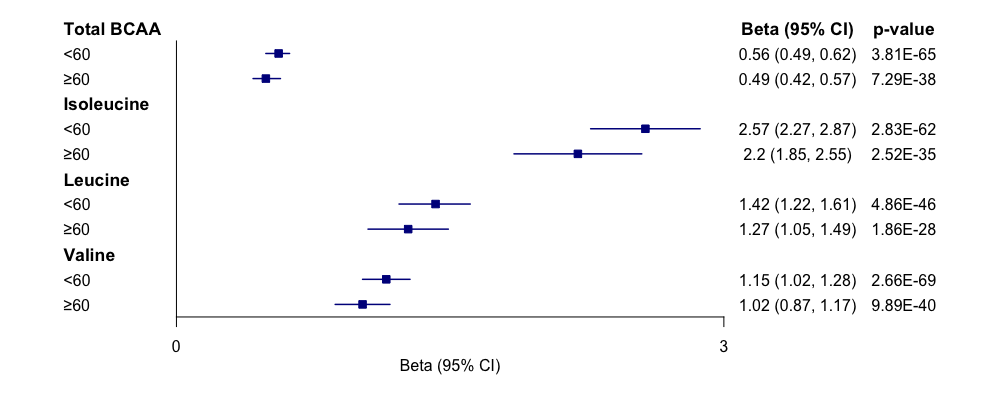 |
| --- | --- |
| b | 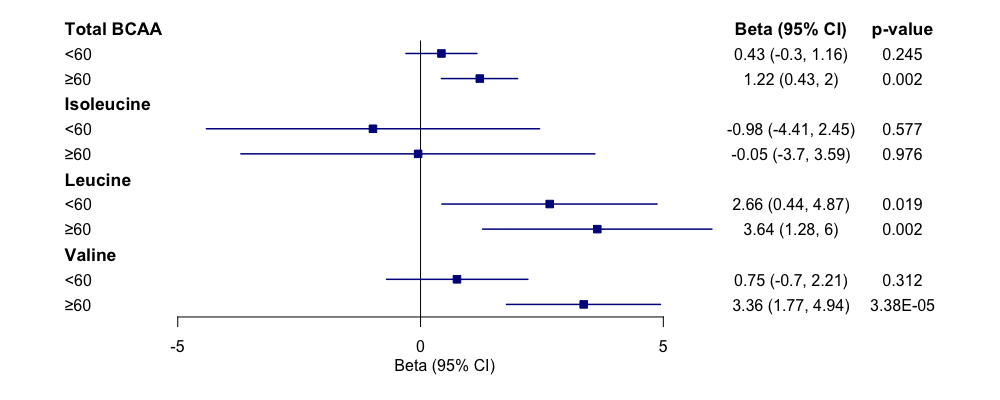 |

**eFigure 1** **Age-specific analyses of associations between circulating BCAAs and ALM/H^2^ (a) and HGS (b).** The estimated change in ALM/H^2^ and HGS per unit increase in circulating BCAAs was represented by beta coefficients (95% CI). Model adjusted age, gender, waist circumference, smoking status, alcohol drinking status, physical activity, ethnicity, education score, and Townsend Deprivation Index. CI, confidence interval; total BCAA, total branched-chain amino acid; ALM/H^2^, appendicular lean mass divided by height squared; HGS, hand grip strength.

| a |  |
| --- | --- |
| b |  |

**eFigure 2** **Sex-specific analyses of associations between circulating BCAAs and ALM/H^2^ (a) and HGS (b).** The estimated change in ALM/H^2^ and HGS per unit increase in circulating BCAAs was represented by beta coefficients (95% CI). Model adjusted age, gender, waist circumference, smoking status, alcohol drinking status, physical activity, ethnicity, education score, and Townsend Deprivation Index. total BCAA, total branched-chain amino acid; ALM/H^2^, appendicular lean mass divided by height squared; HGS, hand grip strength; CI, confidence interval.

a

b

**eFigure 3 Sensitivity analysis**

The estimated changes in ALM/H^2^ (a) and HGS (b) per unit increase in circulating BCAAs are represented by beta coefficients (95% CI) after excluded individuals with age-related chronic diseases (Alzheimer's Disease, Parkinson's Disease, Osteoporosis, Arthritis, and Type 2 Diabetes). Model 1 was adjusted for age, gender, and waist circumference, and model 2 was additionally adjusted for confounding factors such as smoking status, alcohol consumption status, physical activity, ethnicity, education score, and Townsend Deprivation Index. The primary findings were derived from model 2. CI, confidence interval; total BCAA, total branched-chain amino acid; ALM/H^2^, appendicular lean mass divided by height squared; HGS, hand grip strength.
